# Immune adaptor SKAP1 acts a scaffold for Polo-like kinase 1 (PLK1) for the optimal cell cycling of T-cells

**DOI:** 10.1038/s41598-019-45627-9

**Published:** 2019-07-18

**Authors:** Monika Raab, Klaus Strebhardt, Christopher E. Rudd

**Affiliations:** 1 Department of Obstetrics and Gynaecology, School of Medicine, J.W. Goethe-University, Theodor-Stern-Kai 7, 60590 Frankfurt, Germany; 2 Cell Signaling Section, Department of Pathology, Tennis Court Road, University of Cambridge, CB2 1Q Cambridge, UK; 3 Centre de Recherch-Hopital Maisonneuve-Rosemont (CR-HMR), Montreal, Quebec, H1T 2M4 Canada; 4 Département de Medicine, Université de Montréal, Montreal, Quebec, H3C 3J7 Canada

**Keywords:** Immunology, NF-kappaB, Cell signalling

## Abstract

While the immune cell adaptor protein SKAP1 mediates LFA-1 activation induced by antigen-receptor (TCR/CD3) ligation on T-cells, it is unclear whether the adaptor interacts with other mediators of T-cell function. In this context, the serine/threonine kinase, polo-like kinase (PLK1) regulates multiple steps in the mitotic and cell cycle progression of mammalian cells. Here, we show that SKAP1 is phosphorylated by and binds to PLK1 for the optimal cycling of T-cells. PLK1 binds to the N-terminal residue serine 31 (S31) of SKAP1 and the interaction is needed for optimal PLK1 kinase activity. Further, siRNA knock-down of SKAP1 reduced the rate of T-cell division concurrent with a delay in the expression of PLK1, Cyclin A and pH3. Reconstitution of these KD cells with WT SKAP1, but not the SKAP1 S31 mutant, restored normal cell division. SKAP1-PLK1 binding is dynamically regulated during the cell cycle of T-cells. Our findings identify a novel role for SKAP1 in the regulation of PLK1 and optimal cell cycling needed for T-cell clonal expansion in response to antigenic activation.

## Introduction

Polo-like kinase 1 (PLK1) is a serine/threonine kinase that regulates the mitosis of mammalian cells. It is a member of the Polo-like serine/threonine kinase (PLKs) family that are structurally conserved from yeast to mammals. Structurally, they are comprised of a kinase domain at the N-terminus as well as polo-box domain at the C-terminus^1,2^. There are five different PLK family members expressed in mammalian cells where they regulate the cell cycle^2^. PLK1-deficient mice show early embryonic lethality with a failure to progress beyond the 8-cell stage^3^. PLK1 is expressed by primary T-cells^4^ and regulates multiple stages of mitosis^4,5^. In this context, PLK1 expression varies with different stage of the cell cycle, accumulating to maximal levels during G2 and M phases^5–7^. At mitosis, PLK1 activates phosphatase Cdc25C, a positive regulator of Cdc2/Cyclin B1 complex^7^. Cdc2/Cyclin B1 in turn is a cyclin-dependent kinase that regulates G2/M phase transition^8^. Beyond this, PLK1 also contributes to the mitotic exit by regulating the anaphase-promoting complex (APC)^9^. It is localized at centrosomes during inter-phase and prophase but is found at the kinetochore during pre-metaphase and metaphase. During anaphase, PLK1 relocates to spindles, and in the mid-body during telophase^6,10^. These different locations correlate with distinct functions for PLK1 during mitotic progression. PLK1 can directly promote entry into mitosis by activating Cdc25C and Cdk1/Cyclin B1, while increasing microtubule nucleation with the phosphorylation of Nlp, Kizuna and Asp^11^. In addition, PLK1 regulates kinetochore functions and the spindle assembly checkpoint by an interaction with other distinct proteins, INCENP, Bub1 and BubR1^12–14^. Further, PLK1 is needed for cytokinesis due to its regulation of Ect2 and RhoA^15^.

Importantly, the altered expression of PLK1 can induce defects in mitosis that result in aneuploidy and tumorigenesis^16–19^. Different cancer cell lines carry missense mutations of PLK1^20^. In one case, mutations in the C-terminal PBD act to suppress the interaction between PLK1 and HSP90^20^. PLK1 is expressed in cutaneous T-cell lymphomas (CTCLs)^21^ where its down-regulation promotes cell cycle arrest and apoptosis^22^.

Immune cell adaptors also regulate T-cell proliferation and function^23,24^. SLP-76 (SH2 domain containing leukocyte protein of 76 kDa) is one such adaptor that is needed for phospholipase C*γ*1 (PLC*γ*1) activation, calcium mobilization and thymic differentiation^25,26^. It has an N-terminal sterile-*α* motif (SAM) and a carboxy-terminal SH2 domain that binds to adhesion and degranulation-promoting adapter protein (ADAP)^27–31^ and the hematopoietic progenitor kinase-1 (HPK-1)^31^. The C-terminal SH2 domain SLP-76 binds to the ADAP^27,28,32^, while ADAP in turn binds to SKAP1^32,33^. SKAP1 is an adaptor with a unique N terminus, a PH domain and a C terminal SH3 domain^33^. SKAP1 SH3 domain binds to proline residues in ADAP while the ADAP-SH3-like domain binds to SKAP1^34,35^. SKAP1 couples the TCR to the activation of LFA-1^35–40^. SKAP1 regulates RapL-Rap1 binding induced by antigen-receptor ligation^41–46^ and is also connected to Rap1-RIAM^35,47^.

In this study, we show that PLK1 phosphorylates and binds to SKAP1, an interaction that is cell cycle dependent during mitosis. Further, we show that SKAP1 regulates PLK1 kinase activity and that the binding between SKAP1 and PLK1 is needed for optimal T-cell cell division. Our findings identify a novel role for SKAP1 as a scaffold for PLK1 in the regulation for the cell cycle of T-cells.

## Materials and Methods

### Cell culture

Jurkat cells were obtained from the American Type Culture Collection (ATCC), while the T8.1 mouse hybridomas were cultured in RPMI 1640 media containing 10% fetal calf serum (FCS). 293T and Hela cells were grown in DMEM media. Both RPMI 1640 and DMEM media were supplemented with 2 mM L-glutamine and penicillin/streptomycin.

### Antibodies

Anti-mouse CD3 (145-2C11) was from American Type Culture Collection while anti-GFP and anti-GST were from Santa Cruz. Anti-SKAP1 (BD Transduction Laboratories), anti-Myc (Cell Signaling), anti-V5 (Invitrogen), anti-FLAG and anti-β-Actin (Sigma) were purchased as assigned. Mouse monoclonal antibodies used in blotting included PLK1 (1:1,000; Cell Signaling), CDK1 (1:1,000; Millipore), Cyclin B1 (1:1,000; Sigma), Cyclin A (1:1000; Cell Signaling), β-Actin (1:200,000; Sigma), phospho-Histone H3 (S10) (1:1,000; Millipore). HRP-conjugated secondary antibodies (1:5,000) were purchased from the The Jackson Laboratory (Maine). Recombinant kinases PLK1, PLK3, CDK1, CDK2, MAPK, Aurora B, CAMK and ZAP-70 were from ProQuinase. Histone H1 protein were obtained from Biolabs).

### Constructs and transfection

Full-length and fragments of human SKAP1 cDNA were sub-cloned into the pGEX vector as described (Raab *et al*.^42^). These included full-length SKAP1, N-terminal (N-SKAP1; residues 1–104), SK region (SK; residues 209–285), or N plus PH and SK regions (N-PH-SK; residues 1–285) were inserted into a pGEX5x-3 (GE Healthcare) as well as cloned into a Flag-tagged (3 times tag) and an EGFP-tagged pcDNA3.1-Hygro (Invitrogen) vector. Site-directed mutagenesis was employed using QuickChange and Pfu Ultra II Fusion HS DNA Polymerase (Stratagene). The sequence of constructs was each confirmed by in house sequencing (Frankfurt). The PLK1 constructs were then inserted into the 3xFlag-tagged vector. Transfections of Jurkat and T8.1 mouse hybridomas were subsequently performed by electroporation using a BTX ECM 830 electroporator. Transfected cells were transferred to prewarmed complete medium and cultured for 24 hours of recovery. Transfection of 293T cells were performed with Jet Pei according to the manufacturer’s instructions.

### *In situ* proximity analysis

DuolinkTM was used to conduct *in situ* proximity analysis with DuolinkTM *in situ* PLA reagents as described previously^44^. Briefly, Duolink Blocking was followed by the use of anti-PLK1 and anti-SKAP1 and isotype specific secondary antibodies in Antibody Diluent^42^. Duolink ligation with ligase was followed by amplification with Duolink Amplification stock and polymerase at 37 °C.

### Immunoprecipitation and blotting

Precipitation was conducted by solublization of cells in Triton X-100, antibody incubation and precipitation with protein G-Sepharose beads, as described^48–50^. Precipitates were run on SDS-PAGE followed by a transfer to nitrocellulose for immunoblotting^41^. Horseradish peroxidase-conjugated rabbit anti-mouse antibody was used together with enhanced chemiluminescence (Amersham Biosciences) for detection of transferred proteins.

### GST pull down assay

GST-proteins were expressed in a standard manner in *Escherichia coli* BL21 cells with 1 mM isopropyl β-D-1-thiogalactopyranoside (IPTG), as described^42^. Cell Lytic B protocol was used to purify the GST-fusion proteins (Sigma #B7435).

### Kinase assay

*In vitro* kinase assays were performed using 10x PK buffer (New England Biolabs) supplemented with 0.05 mM ATP and 1 μATP and *γ*^32^-ATP (3,000 Ci/mmol; Amersham Pharmacia) for 30 min at 37 °C in the presence of recombinant human active kinase (ProQuinase) and bacterially expressed purified GST proteins as substrates.

### Construction of SKAP1 shRNA vectors

The template for human-mouse–SKAP1 shRNAs was generated by ligating the annealed primers 5′-ACCTCACATTGGACAGGACAGCTCTGTCAAGAGCAGA GCTGTCCTGT CCAATGTTT-3′ and 5′-CAAAAAACATTGGACAGG ACAGCTCTGCTCTTGACA GAGCTGTCC TGTCCAATGTG-3′ (for W1 and Z1), and 5′-CCTCATAACGTAATCAA GCAAG GATTCAAGAGAT CCTTGCTTGATTACGTTATTT-3′ and 5′-CAAAAAAT AACGTAATCAAGCAAGGATCTCTTGAATCCTTGCTTGATTACGTTATG-3′ (for W3 and Z3) into the BbsI sites of psiRNA-hH1 vectors (InVivo Gen). As a control, the primers 5′-ACCTCGCGTTAATTAGACTGAGGA GTTCAAGAGAC TCCTCAGTC TAATTAACGCTT-3′ and 5′-CAAAAAGCGTTAATTAGAC TGAGGAGTCTCTTG AACTCCTCAGTCTAATTAACGCG-3′ were used. Designed RNA oligonucleotides were blasted against the GenBank/EMBL/DDBJ database to ensure gene specificity. This nonrelevant control had no homology with mouse SKAP1 genes.

### Synchronization and cell-cycle distribution by FACS analysis

For cell cycle studies, cells were incubated in the presence of 2 mM thymidine (Sigma; 16 h), released in fresh medium (8 h) and then subjected to a second thymidine block (16 h) as described previously^17^. For FACs, cells were collected after every 3 h to assess changes in cell-cycle kinetics. Cell-cycle quantification of flow cytometry data (FACScan, BD Biosciences) was conducted using ModFit (Verity Software House). 150 nM Nocodazol (Sigma) for 16 hours was used to arrest cells in mitosis.

## Results

### PLK1 phosphorylates N-terminal sites on SKAP1

**Figure 1:**
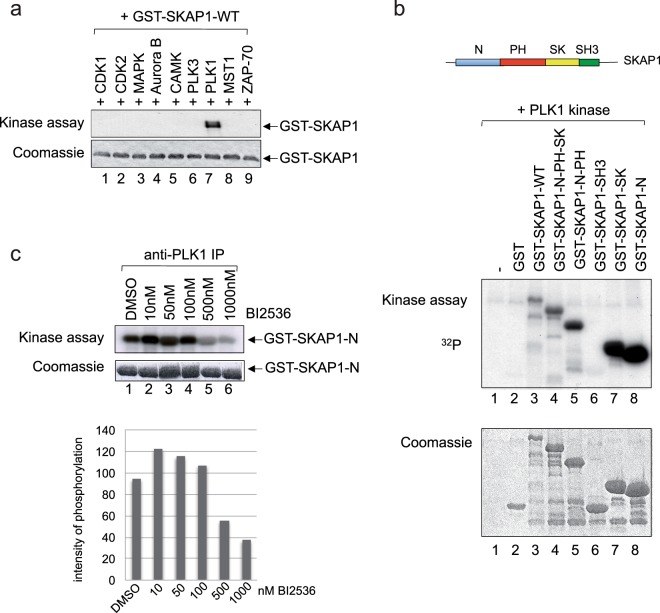
PLK1 phosphorylates the N terminal, PH and SK regions of SKAP1. (Panel a) PLK1 phosphorylates SKAP1. Purified GST-SKAP1 was added to purified recombinant kinases that included CDK1, CDK2, MAPK, Aurora B, CAMK, PLK3, PLK1, MST1 and ZAP-70 in the presence of radio-active phosphate followed by an assessment of phosphorylation on SDS-PAGE (upper panel). Coomassie blue staining of GST-SKAP1 (lower panel). (Panel b) PLK1 phosphorylates the N terminal, PH and SK regions of SKAP1. As above, incubation with fusion proteins of various regions of SKAP1 with recombinant PLK1 in the presence of *γ*^32^-ATP. Coomassie blue staining of the various GST-SKAP1 constructs. (Panel c) BI2536 inhibits PLK1 phosphorylation of N terminal SKAP1. Jurkat T cells were incubated with increasing amounts of the PLK1 Inhibitor BI2536 for 24 hours, followed by immunoprecipitation using anti-PLK1 and *in vitro* kinase assay with GST-SKAP1-N-terminal domain.

To assess a connection between serine/threonine kinases and SKAP1, an array of kinases was tested for an ability to phosphorylate SKAP1 (Fig. 1a). Purified GST-SKAP1 was added to precipitates of individual kinases that included CDK1, CDK2, MAPK, Aurora B, CAMK, PLK3, PLK1, MST1 and ZAP-70 in the presence of radio-active phosphate followed by an assessment of phosphorylation on SDS-PAGE. From this, we observed that PLK1 was the only kinase to phosphorylate the GST-SKAP1 (lane 7). Coomassie Blue staining showed the presence of equal amounts of GST-SKAP1 (lower panel). To map the site of phosphorylation, purified GST-fusion proteins of different regions of SKAP1 and SKAP1 full-length WT were incubated with purified PLK1 kinase in an *in vitro* phosphorylation assay (Fig. 1b). The GST fusion proteins included full-length SKAP1, N-terminal (N-SKAP1; residues 1–104), SK region (SK; residues 209–285), or N plus PH and SK regions (N-PH-SK; residues 1–285). From this, all proteins with the N-terminus or the SK region of SKAP1 were phosphorylated by PLK1 (middle panel). By contrast, PLK1 failed to phosphorylate the GST-SKAP1 SH3 domain. Coomassie Blue staining of the gels confirmed the presence of the GST fusion proteins (lower panel). Further, the small molecule PLK1 inhibitor BI2536 inhibited the phosphorylation of GST-SKAP1 N in anti-PLK1 precipitates from Jurkat T-cells by as much as 60% (lower panel) (Fig. 1c). These data showed that PLK1 can mediate the *in vitro* phosphorylation of several regions in SKAP1.

**Figure 2:**
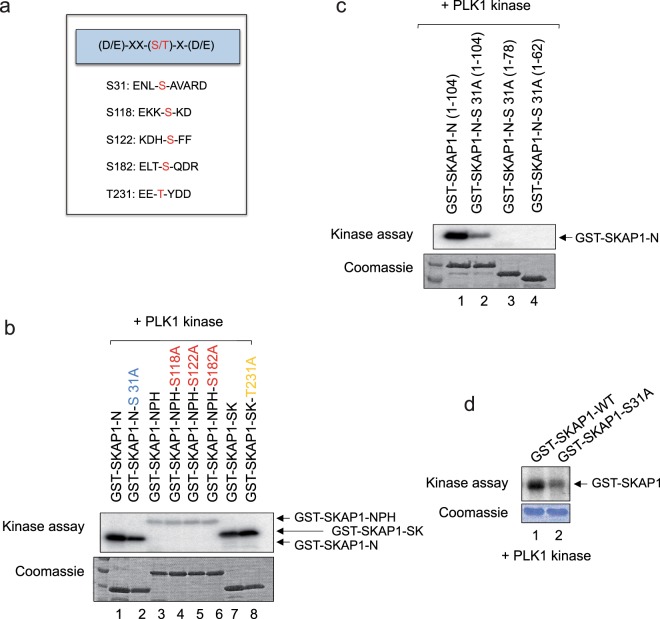
PLK1 phosphorylates the S31 site in SKAP1 N-terminal region. (Panel a) Sequence of SKAP1 showing putative PLK1 phosphorylation sites. (Panel b) PLK1 phosphorylates GST- SKAP1-S31. Purified GST-fusion proteins of different regions in SKAP1 were incubated with purified recombinant PLK1 kinase in an *in vitro* phosphorylation assay. The GST fusion proteins included SKAP1, N-terminal (N-SKAP1; residues 1–104), N-SKAP-S31A, SK region (SK; residues 209–285), SK-T231A or N plus PH; N-PH S118A, N-PH S122A, N-PH, S182A. (Panel c) PLK1 phosphorylates GST-SKAP1-S31A. GST-fusion proteins as outlined were incubated with purified PLK1 kinase in an *in vitro* phosphorylation assay. Panel d: Full-length GST-SKAP1-WT and S31A mutant were incubated with recombinant PLK1 kinase in an *in vitro* phosphorylation assay. Coomassie blue staining shows equal loading.

We then identified potential phosphorylation sites based on the PLK1 consensus sequence (D/E-XX-(S/T)-X-(D/E) in human SKAP1 (Johnson *et al*. 2007; Nakajima *et al*. 2003) (Fig. 2a). Sites include S31: ENL-S-AVARD; S118: EKK-S-KD; S122: KDH-S-FF; S182: ELT-S-QDR and T213: EE-T-YDD. To examine potential sites, full-length and N-terminal fragment of (residues 1–104) human SKAP1 cDNA were sub-cloned into the pGEX vector as described^42^. GST fusion protein wild-type SKAP1 and N-terminal deletion mutants were then used in an *in vitro* kinase assay with PLK1 (Fig. 2b). Mutation of residue 31 in the N- terminal domain (i.e. S31A) of SKAP1 reduced phosphorylation relative to N-terminal wild-type SKAP1 (lanes 1–2). By contrast, mutation of residues S118A, S122A and S182A within the GST-SKAP1-NPH domain construct had no effect on its phosphorylation (lanes 3–6). GST-SKAP1-NPH fusion proteins were less well phosphorylated than the N-domain protein. The phosphorylation of T231A mutant in the SK domain was unaffected relative to wild-type GST-SKAP1 SK (lanes 7, 8). Similarly, a GST-SKAP1-N fusion protein was phosphorylated (Fig. 2c), while mutation of S31A reduced this phosphorylation (lane 2 vs 1). Constructs of residues 1–78 or 1–62 containing the S31A mutant were resistant to phosphorylation (lanes 3, 4). Lastly, the GST-SKAP1-WT-S31A underwent less phosphorylation than GST-SKAP1-WT in an *in vitro* kinase assay (Fig. 2d). Commassie staining showed the presence of equal amounts of the fusion proteins. Overall, these observations indicated that serine 31 was a key phosphorylation site in SKAP1 for PLK1.

### SKAP1 N-terminus binds to the PLK1 kinase domain

**Figure 3:**
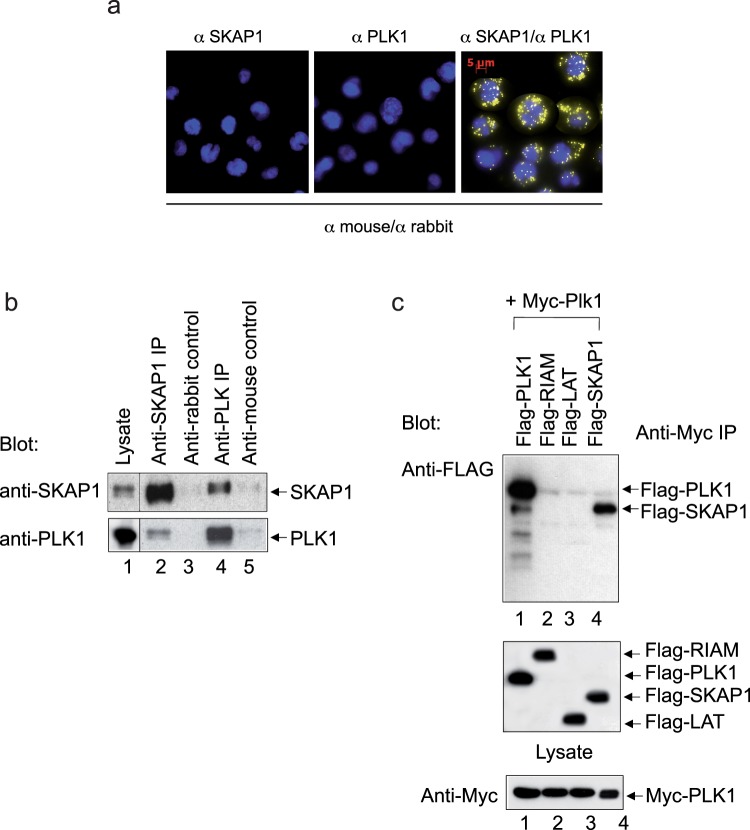
PLK1 binds to SKAP1. (Panel a) Proximity ligation assay (PLA) of endogenous PLK1 and SKAP1 in Jurkat T-cells shows a positive signal. (Panel b) Anti-PLK1 co-precipitates SKAP1 and vice versa. Lane 1: lysate; lane 2: anti-SKAP1; lane 3: anti-rabbit control; lane 4: anti-PLK1; lane 5: anti-mouse control. (Panel c) Anti-PLK1 co-precipitates SKAP1 from transfected Hela cells. Hela cells were transfected with either PLK1, RIAM, LAT or SKAP1 tagged with FLAG in combination with Myc- tagged PLK1. Anti-Myc co-precipitated SKAP1, but not the RIAM or LAT controls (lane 4 vs. 2, 3). It also co-precipitated Flag-PLK1 indicating that it can bind to itself (lane 1). Lower panels show the expression controls for various expressed proteins.

To assess whether PLK1 binds to SKAP1, we first ran a proximity ligation assay (PLA) of endogenous proteins in Jurkat cells to determine whether the two proteins localize in close proximity (Fig. 3a). This assay showed that anti-PLK1 and anti-SKAP1 generated positive signals indicative of a close association, no signal was detected when both antibodies were used alone. Similar PLA signals have been observed between other proteins in the lab previously^44^. Consistent with this observation, anti-SKAP1 co-precipitated endogenous PLK1 from Jurkat T-cells (Fig. 3b, lane 2) ad anti-PLK1 co-precipitated SKAP1 (lane 4). Further, Hela cells were co-transfected with either PLK1, RIAM, LAT or SKAP1 tagged with FLAG in combination with Myc-tagged PLK1 (Fig. 3c). Anti-Myc co-precipitated SKAP1, but not the RIAM or LAT controls (lane 4 vs. 2, 3). It also co-precipitated Flag-PLK1 indicating that it can bind to itself (lane 1). Lower panels show the expression controls for various expressed proteins. These data showed that PLK1 can bind to SKAP1 in mammalian cells.

**Figure 4:**
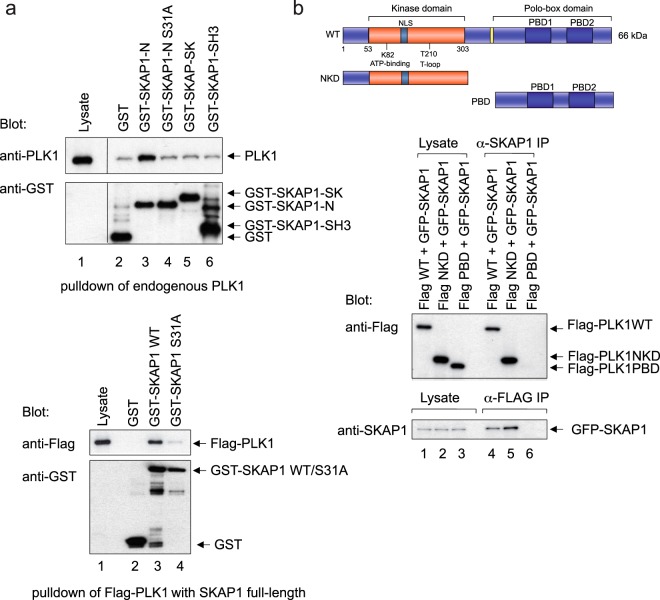
Binding sites between PLK1 and SKAP1. (Panel a) Upper panel::SKAP1 S31 in the N-terminal domain binds PLK1. GST fusion proteins of regions in SKAP1 were used to co-precipitate endogenous PLK1 from lysates of Hela cells. Mutation of S31A in this region disrupted the interaction. Lane 1: lysate; lane 2: GST; lane 3: GST-SKAP1-N; lanes 4: GST-SKAP1-S31A; lane 5: GST-SKAP1-SK; lane 6: GST-SKAP1-SH3. Lower panel: GST full-length proteins of SKAP1 WT and S31A mutant were used to co-precipitate Flag-PLK1 expressed in 293T cells. Lane 1: lysate; lane 2: GST; lane 3: GST-SKAP1 WT; lane 4: GST-SKAP1-S31A. GST-Blot shows equal expression. (Panel b) SKAP1 binds to the NKD kinase domain of PLK1. Co-expression in 293T cells of either Flag-tagged PLK1, N-terminus-kinase domain (NKD) or the PBD domains with GFP-SKAP1. Middle panel: GFP-SKAP1 readily co-precipitated wild type PLK1 (lane 4) and the NKD protein (lane 5), and not to the PBD domains (lane 6). Lower panel: Flag-PLK1 PBD failed to precipitate GFP-SKAP1.

To map the binding regions between the two proteins, GST fusion proteins of regions in SKAP1 were used to co-precipitate endogenous PLK1 from lysates of Hela cells (Fig. 4a, upper panel). The SKAP1 N domain co-precipitated PLK1 (lane 3), and further, mutation of S31A in this region disrupted the co-precipitation (lane 4). Neither the SKAP1 SK or SH3 domain was able to co-precipitate PLK1 (lanes 5 and 6, respectively). S31 is conserved in mouse and human SKAP1. Further, mutation of S31 in full-length SKAP1 also disrupted the interaction with PLK1 (Fig. 4a, lower panel, lane 4 vs lane 3). These data indicated that the N terminal region of SKAP1 interacted with PLK1.

Conversely, Flag-tagged PLK1, N-terminus-kinase domain (NKD) or the PBD domains were expressed with GFP-tagged SKAP1 in the cell line, 293T (Fig. 4b). Anti-SKAP1 co-precipitated Flag tagged PLK1 WT (lane 4) and the NKD protein (lane 5), and not to the PBD domains (lane 6). Anti-Flag precipitation of PLK1 PBD also failed to precipitate GFP-SKAP1. These data indicated that the N terminal region of SKAP1 interacted with the PLK1 kinase domain.

### SKAP1 binding activates PLK1 kinase activity

**Figure 5:**
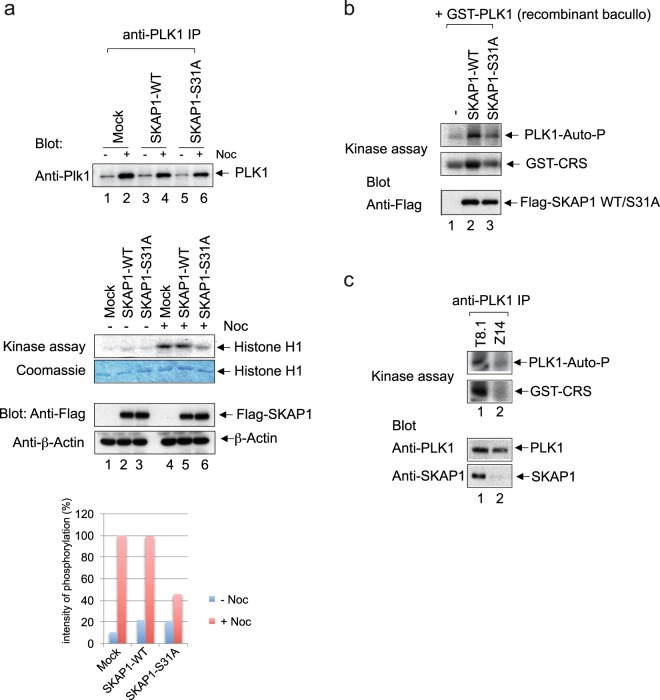
SKAP1 binding regulates PLK1 activity. (Panel a) SKAP1 WT or binding mutant S31A and PLK-1 were co-expressed in Hela cells. Cell growth was arrested in mitosis using 150 nM nocodazole for 16 hours followed by a measure PLK1 activity. Upper panel: Nocodazol induced a similar increase in the expression of PLK1 in cells expressing vector (Mock), WT and S31A SKAP1. Middle panel: Precipitation of PLK1 followed by a measure of *in vitro* kinase activity of PLK1 using the phosphorylation of histone 1 as a target substrate. Coomassie blue staining show equal loading. PLK1 kinase activity in cells expressing the S31A mutant was reduced (lane 6 vs. 4, 5). Middle panel: a similar level of SKAP1 WT and S31A was expressed in the cells. Lower panel: Histogram showing intensity of histone 1 phosphorylation in %. (Panel b) Recombinant PLK1 kinase was incubated with purified Flag-SKAP1 WT and S31A mutant expressed in 293 T and GST-CRS (cytosolic retention signal of Cyclin B1) as substrate for 30 min, 37 °C in the presence of *γ*^32^p-ATP. Anti-Flag blot shows equal expression of FLAG-SKAP1 and S31A mutant. PLK1 auto-phosphorylation and GST-CRS phosphorylation was reduced in the presence of S31A mutant. (Panel c) Down-regulation of SKAP1 expression in mouse hybridoma T 8.1 cells (Z14) shows reduced PLK1 kinase activity. PLK1 was immunoprecipitated from detergent lysates of T8.1 cells and used in an *in vitro* kinase assay with GST-CRS. Anti-PLK1 immunoblot which shows similar levels of immunoprecipitated PLK1. Anti-SKAP1 blot shows down-regulation of SKAP1.

Given this interaction, an important question was whether SKAP1 binding might affect PLK1 kinase activity (Fig. 5). To assess this, SKAP1 full-length WT or the binding mutant S31A were co-expressed with PLK1 in Hela cells. Cell growth was arrested in mitosis using an inhibitor of microtubule dynamics nocodazole (Noc) followed by an assessment of PLK1 activity (Fig. 5a). Cells arrested in mitosis by Noc showed a similar level of PLK1 expression in cells expressing vector (Mock), WT and S31A SKAP1 (upper panel). Despite this, the precipitation of PLK1 followed by a measure of *in vitro* kinase activity of PLK1 using the phosphorylation of histone 1 as a target substrate showed a >50% reduction in PLK1 kinase activity in cells expressing the S31A mutant (middle panel, lane 6 vs. 4, 5 and lower histogram). A similar level of SKAP1 WT and S31A was expressed in the cells as well as beta actin as an additional control (lower panel, lanes, 2, 3, 5 and 6). These data showed that SKAP1 binding to PLK1 is needed to increase in PLK1 activity in mitotic cells.

We further used purified recombinant GST-PLK1 from baculoviral expression with precipitated Flag-SKAP1 full-length WT, or the S31A mutant, in the presence of GST-CRS, the cytosolic retention sequence of Cyclin B1, as a substrate, in an *in vitro* kinase assay (Fig. 5b). While SKAP1 WT increased the kinase activity of recombinant PLK1, the S31A mutant failed to activate PLK1 autophosphorylation or GST-CRS phosphorylation. These data further demonstrated that SKAP1 residue S31 binding to PLK1 was needed for the activation of PLK1.

Next, we performed anti-PLK1 immunoprecipitations from T8.1T WT-cells and Z14 cells where SKAP1 had been knocked-down using shRNA^51^ (Fig. 5c). While anti-PLK1 showed PLK1 activity as seen by its auto-phosphorylation and phosphorylation of GST-CRS, PLK1 phosphorylation of itself and substrate was markedly reduced in Z14 cells (middle panels). As controls, blotting with anti-PLK1 showed equal level of expression, while blotting with anti-SKAP1 showed the loss of SKAP1 expression in the Z14 cells (lower panels). These data confirm further that SKAP1 expression is needed for optimal PLK1 activation.

### SKAP1 interacts with PLK1 during mitosis

**Figure 6:**
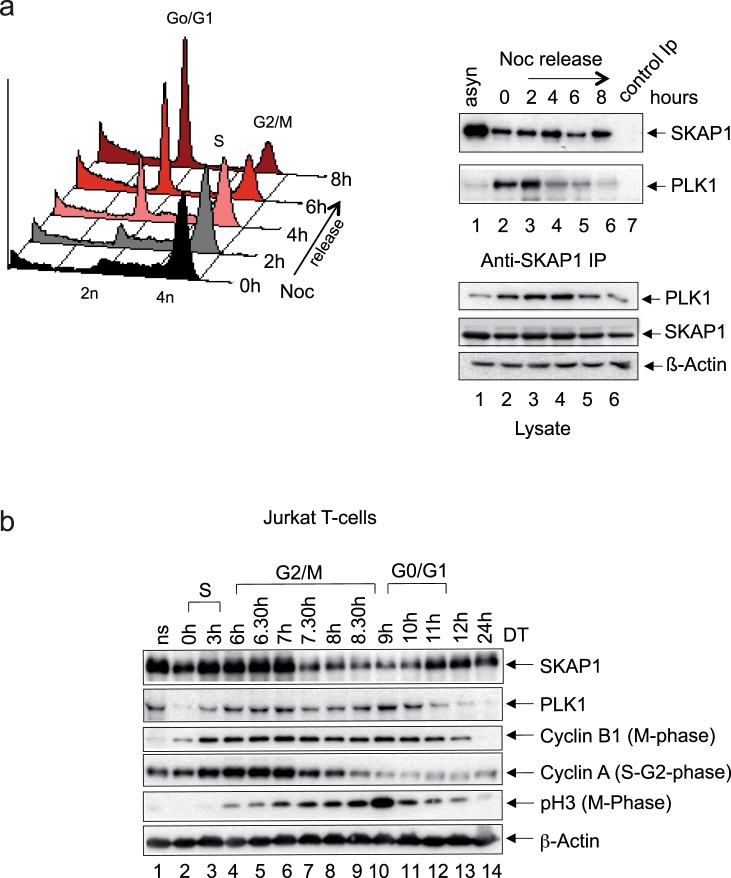
Interaction of SKAP1 and PLK1 is regulated following arrested mitosis. (Panel a) Jurkat T-cells were incubated with 150 nM nocodazole for 16 hours and released in fresh media for 8 hours and analyzed by FACS and Western blotting. Mitotic arrested cells at 0 hour showed a 4N status in G2/M (left panel). Washing the cells and their release from blockade was followed by the progression to 2N in G0/G1 over 2–8 hours. Right panel: Anti-SKAP1 co-precipitation of PLK1 with SKAP1 following release from mitotic arrest. Lower panels: Blotting of cell lysates with anti-PLK1, SKAP1 and β-Actin. (Panel b) Jurkat cells were synchronized by double thymidine treatment and analyzed by Western blotting with antibodies against SKAP1, PLK1, Cyclin B1, Cyclin A, pHistone, H3 and β-Actin.

Given the role of PLK1 in regulating cell division and the ability of SKAP1 to influence PLK1 activity, we next assessed whether the binding of PLK1 to SKAP1 occurs during and following mitosis. Jurkat T-cells were incubated with Noc to synchronize the cells in mitosis (Fig. 6). Most of the blocked cells had a 4N status in G2/M, while the release was followed by the progression to 2N in G0/G1 over 2–6 hours (Fig. 6a, left panel). Precipitation with anti-SKAP1 from cell lysates following the release showed the co-precipitation of PLK1 with SKAP1 from 0–2 hours (right upper panels). Anti-SKAP1 co-precipitated PLK1 from 0–2 hours post-release followed by a progressive reduction from 4–8 hours. As a control, the blotting of cell lysates showed a sustained expression of PLK1, SKAP1 and β-Actin over this time frame (lower panels). PLK1 expression was highest from 0–6 hours. As a further control, we monitored the expression of SKAP1, PLK1 regulators of cell cycle at various stages of mitosis (Fig. 6b). SKAP1 was expressed at its highest level during S and early G2/M and late G0/G1, while PLK1 was expressed mostly during G2/M and G0/G1. Cyclin B1 was expressed mostly during M-phase and Cyclin A during S-G2 and pH3 during M-phase as expected. Overall our data showed that SKAP1 expression and its binding to PLK1 is dynamically regulated during the cell cycle of T-cells.

### T-cells with reduced SKAP1 showed a delayed onset of G2/M

**Figure 7:**
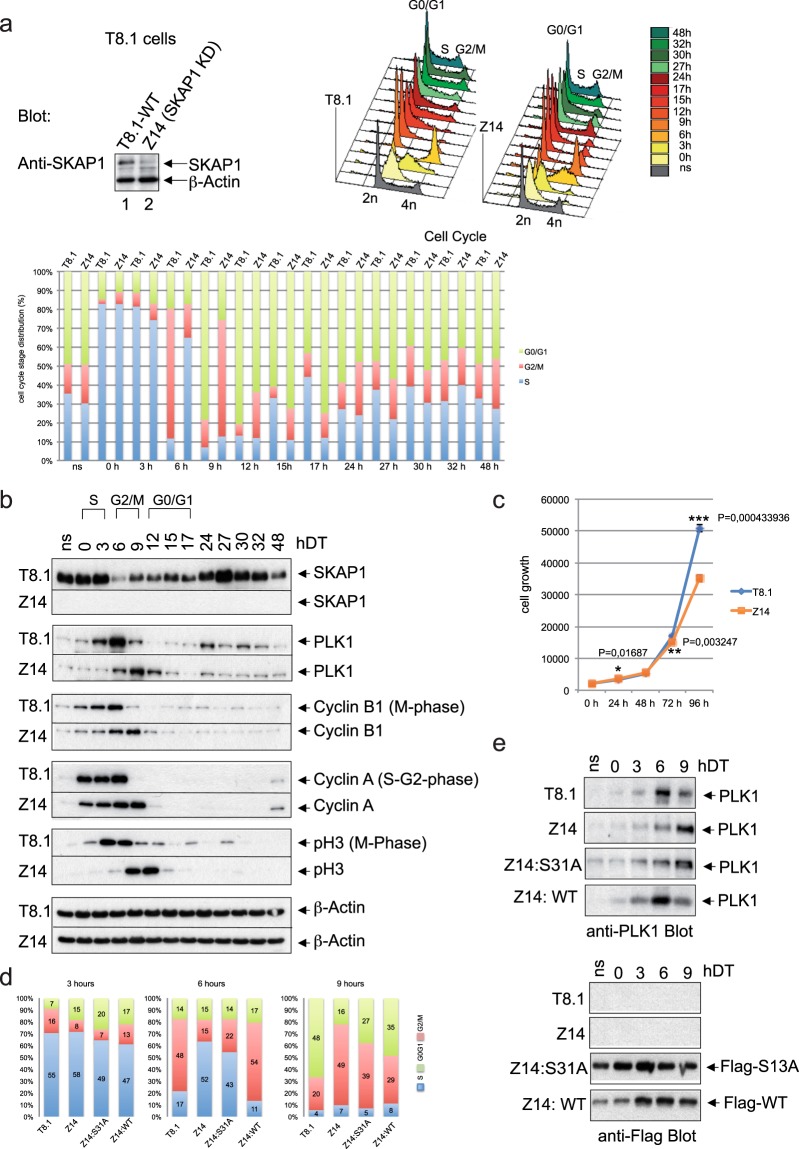
SKAP1 regulates cell cycle of T-cells. (Panel a) SKAP1 shRNA knock-down of T-cells delays T-cell cycle progression. Cells were subjected to double thymidine (DT) labelling to monitor the kinetics of cell cycle progression. Upper left inset: shows down-regulation of SKAP1 expression in T-cells by SKAP1 shRNA. Right upper panel: FACS profile of DT synchronized mouse WT (T8.1) and SKAP1 KD Z14 T cells. Middle lower panel: Histogram showing percentages of cells which were in the G0/G1, G2/M and S phase. (Panel b) SKAP1 shRNA knock-down T-cells show a delay in expression of key regulators or cell cycle progression. The expression of regulators of cell cycle such as Cyclin B1 (M- phase), Cyclin A (S-G2 phase), pH3 (M phase) in wild-type cells versus Z14 cells by immunoblotting of cell lysates. (Panel c) Growth curve of SKAP1 WT T cells and SKAP1 shRNA knock-down T cells over a period of 96 hours. Z14 T cells show a lower cell number relative to T8.1 WT cells. (Panel d) SKAP1 WT, but not SKAP1-S31A, rescues Z14 T-cells for normal cell cycle. Z14 T cells, transfected with either Flag-SKAP1 WT or a S31A mutant, were synchronized by double thymidine treatment and analyzed by FACS and immunoblotting. The expression of Flag-SKAP1 WT restored a normal cell cycle pattern in Z14 cells that was comparable to T8.1 cells. By contrast, expression of S31A mutant fail to restore normal cell cycle. The pattern of PLK1 expression in Flag-SKAP1 WT transfected Z14 T cells was similar to T8.1 WT cells. Flag S31A expression in Z14 showed no effect.

These above data suggested the SKAP1-PLK1 contributes to the efficiency of the cycling of T-cells. Initially, the cycling of shRNA KD Z14 cells were contrasted with wild-type cells^51^ (Fig. 7a, top left inset). Cells were subjected to double thymidine (DT) synchronization to monitor the kinetics of cell cycle progression. Indeed, SKAP1 KD Z14 T-cells showed a slower rate of cell cycle progression (top right inset). While S phase commenced at the same time for both cells post-block (0–3 hours), a delay of 3–6 hours was seen in Z14 cells (lower histogram). Strikingly, by 6 hours, >65% of WT cells had entered in G2/M phase while 12% of Z14 had entered this phase. Z14 cells entered G2/M over a more extended period with 20–25% by 12 h and 18% by 12 hours. Similarly, Z14 cells were delayed in entry to S phase when compared to WT cells. These data showed the reduction in SKAP1 expression retards cell cycle progression in T-cells.

Next, we examined the expression of regulators of cell cycle such as Cyclin B1 (M phase), Cyclin A (S-G2 phase) and phospho-Histone H3 (pH3) (M phase) in wild-type cells versus Z14 cells (Fig. 7b). Consistent with the delay in cell cycling, the expression of the PLK1, Cyclin B1, Cyclin A and pH3 in Z14 cells was delayed by some 3–6 hours relative to wild-type cells. The expression of PLK1 peaked at 3–9 hours in WT cells and at 6–12 hours in Z14 cells. The expression of Cyclin B1 and pH3 of M phase was observed 0–6 hours in wild-type cells and at 6–12 hours in Z14 cells. As a control, no effect was seen on the expression of β-Actin. Consistent with these observations, the reduction in SKAP1 expression reduced significantly the cell growth rate as seen by the reduced numbers of Z14 cells in culture at 72 and 96 hours (Fig. 7c). For example, at 96 hours, there were 30% fewer Z14 cells that WT T-cells. These data further show that SKAP1 promotes the cell cycle progression of T-cells.

Lastly, it was important to examine whether the interaction between SKAP1 and PLK1 controlled the efficacy of cell division (Fig. 7d). For this, we performed rescue experiments by transfecting Z14 cells with either Flag-SKAP1 WT, or the Flag-SKAP1 S31A mutant. Flag-SKAP1 WT expression in Z14 cells was able to rescue the cell cycle progression to that seen in WT cells. By contrast, the Flag-SKAP1 S31A mutant failed to restore the pattern of cell progression seen in WT cells. For example, by 6 hours, 48–55% of WT T8.1 and reconstituted Z14 WT cells entered G2M, while only 15–22% of Z14 or Flag-SKAP1 S31A reconstituted cells had entered by this time. The expression of both constructs was also monitored during the cell cycle after double thymidine block with anti-Flag antibodies (Fig. 7e). Peak expression of PLK1 was seen at 6 hours in the WT and the Z14 expressing SKAP1, and at 9 hours in the Z14 and Z14 cells expressing SKAP1 S31A (upper panel). The expression of transfected WT and Flag-SKAP1 S31A protein was similar during this period (lower panel). Overall, this data shows that the interaction between SKAP1 and PLK1 controls the efficacy of T-cell division.

## Discussion

SKAP1 has previously been shown to activate LFA-1 following antigen-receptor ligation^38,52^. Whether this was the adaptors only function in the activation of T-cells had been unclear. Here, we define a second function for SKAP1 involving the regulation of PLK1 activity and T-cell cycling. We found that SKAP1 was both bound to PLK1, mapped the binding region to the N-terminus-kinase domain of PLK1 and showed that SKAP1 is a substrate of PLK1. Further, we showed that SKAP1 binding regulated PLK1 kinase activity, and lastly, that the SKAP1-PLK1 complex was needed for the optimal cycling and cellular expansion of T-cells. Overall, PLK1 is known to control a number of processes in the cell cycle by phosphorylating different substrates, our findings now identify a novel role for an immune cell specific mediator SKAP1 in the regulation of PLK1 and the cell cycle of T-cells.

We previously showed the SKAP1 could localize to the nucleus of T-cells, and for this reason, searched for nuclear kinases that phosphorylate the adaptor^32^. Remarkably, PLK1 phosphorylated SKAP1 under *in vitro* conditions where related PLK3, and a range of other kinases such as CDK1, CDK2, MAPK, Aurora B, CAMK, and MST1 failed to phosphorylate the substrate. The specificity was noteworthy given that PLK1 and PLK3 are related (>35%) with conserved regulatory sites and a similar overall structure^53^. This suggested a specific kinase-substrate relationship between PLK1 and SKAP1. Further, PLK1 phosphorylated the SKAP1 at serine residue 31 which served as a binding site for PLK1. This suggested the possibility that PLK1 regulates its own binding. It is unclear whether the phosphorylation of SKAP1 S31 site is needed for binding to occur, and whether the binding to SKAP1 is needed for PLK1 to phosphorylate adjacent SKAP-1 molecules. Indeed, SKAP1 can undergo dimer formation^45,46^, and so, PLK1 binding to one subunit could potentially act as a scaffold for the phosphorylation of the second associated subunit within the complex.

It is also noteworthy that the S31 residue is located in a region homologous to a coiled-coil domain in related SKAP-1R (SKAP-related or SKAPHOM)^32,45,54^. We had previously shown that this region binds to RapL, an immune cell isoform of the RASSF5 (Ras association domain family 5) family^55^ and is needed for the regulation of T-cell adhesion and motility^42,43^. Whether the PLK1 binding to SKAP1 competes or complements binding to the RapL complex remains to be determined. However, this is considered unlikely given that although the interactions occur in different regions of the T-cell. SKAP1-RapL binding and its subsequent binding to the cytoplasmic tail of the integrin LFA-1 occurs at the cell surface^42–44^, while SKAP1-PLK1 interacts in the nucleus. The interactions therefore are likely to occur temporally at different times and different locations of the cell. A similar multi-functional role of an immune cell adaptor in different regions of a cell has been seen with SLP-76 which regulates TCR proximal signaling need for calcium mobilization^26,56^ as well as binding to RanGAP1 of the nuclear pore complex for the transport of transcription factors into the nucleus of T-cells^57^.

Our second major finding was that SKAP1 could regulate the kinase activity of PLK1. This effect was seen in kinase assays where the mutation of residue S31 of SKAP1 as well as the down-regulation of SKAP1 by shRNA resulted in a reduction in PLK1 catalytic activity. Activity was measured by PLK1 auto-phosphorylation as well as its phosphorylation of exogenous substrates histone H1 and GST-CRS. Moreover, these effects of SKAP1 down-regulation or the binding mutant were either seen in reconstitution studies in Hela cells, as well as in *in vitro* kinase assays of anti-PLK1 precipitates from WT T8.1 vs. Z14 SKAP1 KD cells. The molecular basis for the activation of PLK1 is unknown but could be direct given that SKAP1 binds to the N-terminus-kinase domain (NKD) of PLK1. The crystal structure of PLK1 has shown that the two Polo box (PB) motifs which form an intramolecular dimer that is joined by two linkers which limits nucleotide hydrolysis^58^. Further structural studies of the SKAP1- PLK1 complex will be needed to clarify mechanism of regulation of PLK1 by SKAP-1 more fully. The binding site of SKAP1 is distinct from the binding sites of proteins like the heat shock protein 90 (HSP90) where mutations abrogate binding in the C-terminal PBD^20^. These different mechanisms enable the complex temporal control of PLK1 activity in cells.

In keeping with the functional importance of SKAP1-PLK1 interaction to cell cycling, the down-regulation of SKAP1 and the expression of the SKAP1 S31A mutant delayed on cell cycle of T-cells. Plk1 controls a number of processes throughout the cell cycle, including centrosome maturation^6^ mitotic entry^59^ chromosome segregation^60^ and cytokinesis^61^ by phosphorylating different substrates. We found that cell cycle progression was postponed with the down-regulation of SKAP1 resulting in a reduction in the T-cell number by some 30% over 96 hours. This was concurrent with a delay in the expression of PLK1, Cyclin A, and pH3. Further, in keeping with the importance of the interaction itself, we found that Flag-SKAP1 S13 mutant expression in Z14 knock-down cells failed to rescue cell cycle progression, while equivalent expression of Flag-SKAP1 WT restored normal cell cycling. SKAP1, therefore, adds to the aggregate signaling needed for T-cell cell cycling. In other words, while the interaction is not essential, it adds to aggregate signaling to make cell cycling more efficient. Due to its restricted expression to immune cells, the SKAP1 pathway appears to have evolved as an additional immune cell specific pathway by which immune cell receptors regulate T-cell division in response to foreign pathogens and cancer neoantigens.

Lastly, our findings define an additional role for SKAP1 as an immune cell adaptor with the capacity to regulate multiple functions in T-cells. SKAP1 regulates PLK1 kinase activity in addition to its role in the regulation of LFA-1 activation by anti-CD3 ligation^35,36,41,42^. It is also the first example of direct SKAP1 regulation of a serine and threonine kinase in T-cells. It is possible that these different functions operate at various stages of T-cell activation and in different subcellular locations in the T-cell. Further, the regulatory effect of SKAP1 on PLK1 occurred in response to signals linked to mitotic arrest. This contrasts with the SKAP1 activation of LFA-1 which occurs directly in response to anti-CD3 ligation^35,36,41,42^. SKAP1 induced LFA-1 activation also depends on the strength of the TCR signal where it has its greatest effect on low affinity or TCR signals^36^. Further, SKAP1 can bind and activate the exchange factor RasGRP1 which, in turn, negatively regulates the p21^ras^-ERK pathway in T-cells^62,63^. In addition, SKAP1 associated ADAP regulates the NFkB pathway^64^. These observations underscore the multi-functional nature of these immune cell specific adaptors in the regulation of T-cell signaling. Further work will be needed to define the exact temporal involvement of this adaptor in regulating various stages on PLK1 involvement in the expansion of T-cells and in different T-cell subsets^65^.
